# A review of generalist and specialist community health workers for delivering adolescent health services in sub-Saharan Africa

**DOI:** 10.1186/1478-4491-11-54

**Published:** 2013-10-26

**Authors:** Adam D Koon, Jane Goudge, Shane A Norris

**Affiliations:** 1 MRC/Wits Developmental Pathways for Health Research Unit, University of the Witwatersrand, Johannesburg, South Africa; 2 Department of Global Health and Development, London School of Hygiene and Tropical Medicine, 15-17 Tavistock Place, London WC1H 9SH, UK; 3 Center for Health Policy, University of the Witwatersrand, Johannesburg, South Africa; 4 Department of Pediatrics, University of Cambridge, Cambridge, UK

**Keywords:** Community health workers, Youth friendly health services, Adolescent health, Sub-Saharan Africa

## Abstract

**Background:**

The health of adolescents is increasingly seen as an important international priority because the world’s one point eight billion young people (aged 10 to 24 years) accounts for 15.5% of the global burden of disease and are disproportionately located in low- and middle-income countries (LMICs). Furthermore, an estimated 70% of premature adult deaths are attributable to unhealthy behaviors often initiated in adolescence (such as smoking, obesity, and physical inactivity). In order for health services to reach adolescents in LMICs, innovative service delivery models need to be explored and tested. This paper reviews the literature on generalist and specialist community health workers (CHWs) to assess their potential for strengthening the delivery of adolescent health services.

**Methods:**

We reviewed the literature on CHWs using Medline (PubMed), EBSCO Global Health, and Global Health Archive. Search terms (n = 19) were sourced from various review articles and combined with subject heading ‘sub-Saharan Africa’ to identify English language abstracts of original research articles on generalist and specialist CHWs.

**Results:**

A total of 106 articles, from 1985 to 2012, and representing 24 African countries, matched our search criteria. A single study in sub-Saharan Africa used CHWs to deliver adolescent health services with promising results. Though few comprehensive evaluations of large-scale CHW programs exist, we found mixed evidence to support the use of either generalist or specialist CHW models for delivering adolescent health services.

**Conclusions:**

This review found that innovative service delivery approaches, such as those potentially offered by CHWs, for adolescents in sub-Saharan Africa are lacking, CHW programs have proliferated despite the absence of high quality evaluations, rigorous studies to establish the comparative effectiveness of generalist versus specialist CHW programs are needed, and further investigation of the role of CHWs in providing adolescent health services in sub-Saharan Africa is warranted.

## Introduction

The World Health Organization (WHO) defines adolescence as the period of time between 10 and 19 years of age, while the United Nations (UN) has historically relied on the term ‘youth’ to describe individuals between 15 and 24 years of age [1,2]. Both terms refer to an age group that is particularly affected by the structural and proximal social determinants of health and development. Structural determinants of adolescent health include national wealth and income inequality, education, war and conflict, and gender and ethnic inequalities. Proximal determinants, by contrast, focus on the circumstances of daily life and can be seen in the school environment, families, neighborhoods, peers, and certain health behaviors. Evidence suggests that structural forces have the strongest impact on adolescents, but that proximal determinants also play a significant role in exposing young people to health-compromising conditions [3].

The health of adolescents is important given the population’s size and burden of disease. Currently, there are one point eight billion young people aged 10 to 24 years, which represents 27% of the global population. Over 90% of this age group is located in low- and middle-income countries (LMICs). Young people aged 10 to 24 account for at least 15.5% of the global burden of disease and, in a given year, an estimated two point six million young people may die from road traffic accidents, self-inflicted injuries, and violence, among other causes [4,5]. Though the adolescent share of burden of disease (15.5%) is proportionately lower than their share of the population (27%), an estimated 70% of premature adult deaths are attributable to health behaviors often begun in adolescence (such as smoking, obesity, and physical inactivity) [2]. Also, adolescent health affects at least five of the Millennium Development Goals (MDG), particularly MDG 5 on maternal health and access to reproductive health as well as MDG 6 on HIV/AIDS, malaria, and other diseases [6].

In 2002, WHO suggested an approach for adolescent health, called Adolescent Friendly Health Services (AFHS) [1]. The Adolescent Friendly Health Services model focuses on providing a package of health services that addresses specific health needs of adolescents in low-, middle-, and high-income countries (see Additional file 1 for characteristics of AFHS). Over time, this package of services has been broadened to include the health needs of young people 10 to 24 years of age and is accordingly called Youth Friendly Health Services (YFHS) [7]. While some programs have experienced success [8], often YFHS are plagued by poor coverage, inadequate implementation, or brief follow-up periods [9,10]. Despite this, there have been calls for YFHS to be brought to scale and to be implemented over a longer period of time [11-13].

Systematic reviews of efforts to increase utilization of adolescent health services in LMICs, reported that the evidence was often limited by weak study design [14,15], such as lack of data on process evaluations, long-term follow-up, cost-effectiveness, scale-up, and the impact on health outcomes. Thus, more research is warranted to establish effective modes of delivering adolescent health services if YFHS is to be adopted in LMICs. Furthermore, new service delivery strategies will need to account for the shortage of health workers that cripple many LMIC health systems [16].

One approach might be to use community health workers (CHWs) to channel facility-based YFHS to households in the wider community. The reasons for this are multiple. First, CHWs have been in existence for at least 50 years and well-established programs exist in many LMICs [17]. Second, strategies to strengthen YFHS do not require advanced technical or clinical training [18]. Third, there exist many types of CHWs [19] and YHFS could potentially draw from the experience of CHWs in different settings, at different times, and with different responsibilities. Fourth, the CHW model focuses on engaging the target population on favorable grounds and in ways that facilitate the uptake of healthy behaviors [20], which is precisely the sensitive community-based approach necessary for YHFS. Fifth, though the WHO framework identifies ‘outreach workers’ as a potential component of adolescent health programs, there is little indication that this actually being implemented in LMICs [18].

To explore these ideas, we reviewed the literature on CHW programs in sub-Saharan Africa. Our objectives were two-fold. First, we determined whether there was evidence to support the delivery of adolescent health services through CHWs in sub-Saharan Africa. Second, we organized the findings based on two broad typologies of CHWs for comparison. This included a review of ‘specialist’ and ‘generalist’ CHWs. We defined ‘specialist’ CHWs as those who have acquired and deployed a narrowly defined set of skills determined by population group (such as, maternal health) or disease (such as, tuberculosis). ‘Generalist’ CHWs, by contrast, have a broader mandate, which attempts to serve the primary healthcare needs of the whole community. As depicted in Table 1, the main differences between specialist and generalist CHWs are the scope of training and job responsibilities.

**Table 1:** Generalist versus specialist community health worker typology

	**Generalist**	**Specialist**
Recruitment	Community involved in identification and selection of potential CHWs	
	Advertisement for candidates through multiple media outlets	
	Criteria: 18 to 40 years of age, from local community, permanent resident, literate, motivated	
Training	Initial: six months	Initial: one to two weeks
	On-the-job: six months	On-the-job: two weeks
	Ongoing: once per month	
	Refresher: every six months	
	Consists of didactic, interactive sessions	Consists of didactic, interactive sessions
	*Core training*	*Core training*
	Access resources, service coordination, crises management, knowledge of health services, leadership, organizational skills, interpersonal communication skills, confidentiality	Promotive, preventive, and therapeutic interventions
Job responsibilities	Many	Few
	Broad	Specific
Monitoring and evaluation	One supervisor per 20 to 25 CHWs	
	*Evaluation*	
	One annual internal evaluation	
	One external evaluation every five years	

Adapated from [19].

## Methods

**Figure 1:**
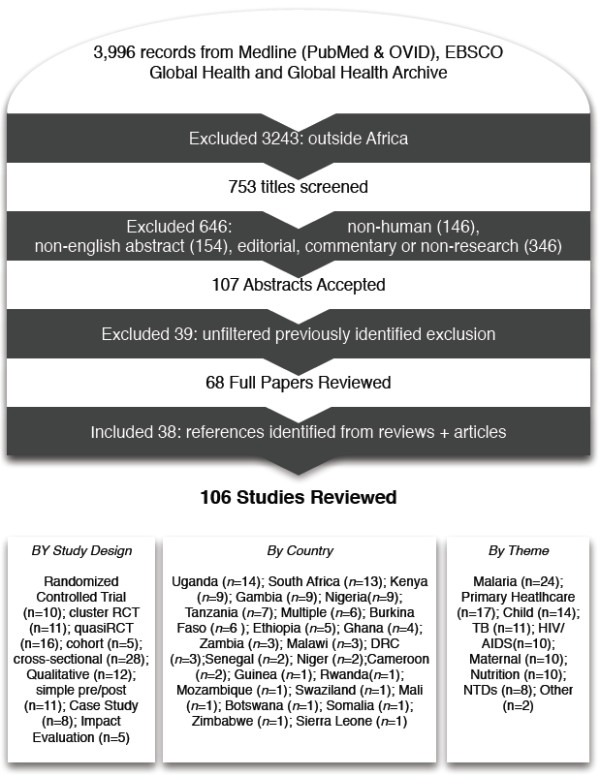
Review flow chart.

A review of the peer-reviewed literature was conducted for original research articles on CHW programs in sub-Saharan Africa (see Figure 1). The following electronic databases were searched in October 2012: PubMed-Medline, EBSCO, Global Health and Global Health Archive (up to October 2012). Additionally, Google and Google Scholar search engines were used to identify sources not included in the electronic databases. Lastly, we made use of five important CHW reviews [17,20-23], particularly WHO’s exhaustive 2010 CHW report [19], for search terms and additional references. All English language abstracts from 1950 to October 2012 were included. For our purposes, we defined a CHW as a lay individual responsible for providing various health services at the community level. Search terms included a combination of the MeSH term “sub-Saharan Africa” with the following: MeSH-“Community Health Worker”, MeSH-“Village Health Worker”, “Lay Health Worker”, “Health Extension Worker”, “Village Health Teams”, “Agentes Polivalentes Elementares”, “Women Group Leaders”, “Village Malaria Worker”, “Nutrition Volunteers”, “Nutrition Worker”, “Community Drug Distributor”, “Village Health Helper”, “Mother Coordinator”, “Village Drug-kit Coordinator”, “Community Reproductive Health Worker”, “Community Mobilizers”, “Health Promoter”, “Peer Counselors”, and “Traditional Birth Attendants”. Commentaries, editorials, dispatches from the field, and news articles were excluded. Publication of multiple trials within the same study was included. We also excluded articles without an evaluative component, studies that focused on livestock CHW programs, CHW programs outside sub-Saharan Africa, and evaluations of knowledge, attitudes, and practices of CHWs.

Data was extracted from selected articles by AK. All authors agreed on the extraction of the following information from each article: year, location, study design, outcome measures, and job responsibilities. Where possible, the reviewing author indicated recruitment criteria, supervision, and training duration of CHWs; although this information was not explicitly reported by many of the included studies. Authors consulted with each other when ambiguities arose between papers. SN repeated the search to verify the first search and included papers. In delineating between generalist and specialist CHWs, the reviewing author judged which characterization appeared to be most appropriate, according to the definitions agreed upon by all authors.

## Results

**Figure 2:**
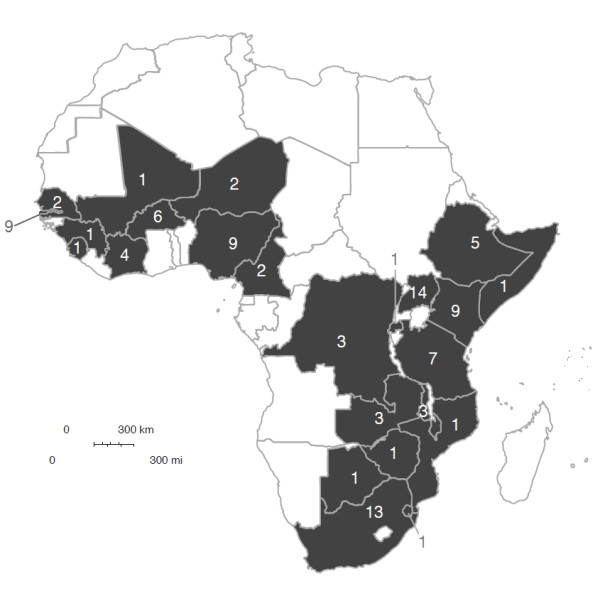
**Countries in sub-Saharan Africa with community health worker (CHW) programs.** This represents the number of CHW articles published in a given country that met our search criteria. We found a wide variety of CHW programs in 24 different African countries.

Sub-Saharan Africa has seen a large number of CHW programs (as shown in Figure 2). A total of 753 articles were returned from our initial search. Applying our exclusion criteria reduced the number of studies to 106. Articles ranged from 1985 to 2012. The number of relevant research articles is rapidly increasing (1980s, n = 15; 1990s, n = 20; 2000s, n = 55; 2010s, n = 16). The literature shows a semantic shift from ‘village health worker’ to ‘community health worker’ over a period of approximately ten years from 1985 to 1995. The term ‘lay health worker’ is used sporadically throughout, but almost exclusively in South Africa.

A wide variety of studies have been conducted in several African countries. These include small, randomized controlled trials (RCT), such as those examining malaria control in The Gambia [24], or cross-sectional analysis of specific components of broader national programs, such as Ethiopia’s Health Extension Worker program [25]. The most common study designs reported in the literature were cross-sectional (n = 21), quasiRCT (n = 15), clusterRCT (n = 11), simple pre/post (n = 11), qualitative (n = 10), and RCT (n = 10). A total of 24 African countries are host to studies identified by our search criteria. The best-represented countries are Uganda (n = 14), South Africa (n = 13), Kenya (n = 9), The Gambia (n = 9), and Nigeria (n = 9). Most programs are in rural areas, but urban CHW programs do exist [26-28].

Many studies are nested within CHW programs, but do not evaluate the CHW component itself. When evaluations were reported, they were not comprehensive and assessed specific services, components, or interventions within the larger program. Similarly, the literature is rich with CHWs acting as field-testing agents for new technologies such as insecticide-treated nets [29,30]. Also, there is a growing presence of nationwide programs in the literature, though no comprehensive evaluations of these programs, outside of the WHO case studies [19], were found.

Many articles focused on the evaluation of the specialized services that CHWs are expected to perform (see Table 2). Whether or not the CHW position was developed specifically for this purpose or these skills have been included for trial purposes, is not always clear. The number of articles for CHWs providing specialized services varied by theme such as malaria (n = 24), child health (n = 14), tuberculosis (n = 11), and nutrition (n = 8). Generalist CHW programs, however, were reported by 17 articles. A single article matched our search criteria that referred to CHWs delivering adolescent health services [31].

**Table 2:** Number of community healthy workers (CHW) articles published by country and specialized CHW service

**Country**	**Number of CHW articles by specialized services**									
	**Malaria**	**PHC**	**Child**	**TB**	**HIV**	**Maternal**	**Nutrition**	**NTDs**	**Other**	**TOTAL**
Uganda	3		2	1	1		1	6		14
South Africa		3	1	4	2	1	2			13
Kenya	2	2	3		1		1			9
Gambia	5	3	1							9
Nigeria	3	3	1				2			9
Tanzania	1		1	1		3			1	7
Multi			1		2		1	2		6
Burkina	2	2				2				6
Ethiopia	1			3		1				5
Ghana		1	2			1				4
Zambia		1	1		1					3
Malawi				1	1	1				3
DRC	1						2			3
Senegal	1						1			2
Niger	2									2
Cameroon	1				1					2
Guinea									1	1
Rwanda	1									1
Mozambique			1							1
Swaziland				1						1
Mali	1									1
Botswana		1								1
Somalia		1								1
Zimbabwe					1					1
Sierra Leone						1				1
TOTAL	24	17	14	11	10	10	10	8	2	106

(HIV, human immunodeficiency virus; NTDs, neglected tropical diseases; PHC, primary health care; TB, tuberculosis).

## Discussion

In addressing our first aim, we found limited evidence on the delivery of adolescent health services through CHWs. The single article by Ross *et al.*, which referred to a large-scale community randomized trial in Tanzania, demonstrated that existing CHWs could help improve knowledge, reported attitudes and behaviors of adolescents on HIV and sexual health [31]. In this study, two to four CHWs attached to a government facility were trained to deliver YFHS in addition to family planning services and case management of sexually transmitted infections. These CHWs were trained for one week and supervised quarterly by eight staff members from a reputable non-governmental organization (NGO). As one of four components of the intervention, however, it is unclear how much of the program’s success was attributable to the involvement of CHWs. In addition to training CHWs to provide YFHS, the project launched community activities, teacher-led, peer-assisted sexual health education in levels 5 to 7 of primary school, and peer condom social marketing. While these additional components likely had a sizable effect on the reported outcomes, the authors maintain that it is possible to effectively deliver adolescent health services through established CHW programs [31]. More research of this caliber should be conducted in different African settings to further validate these findings.

Another key finding is that limited evidence exists on the impact of CHWs in sub-Saharan Africa. Global reviews have described a wealth of mixed evidence from CHWs around the world, but very few high quality studies evaluate the health impacts of CHW programs in African countries. Three randomized controlled trials in The Gambia revealed reductions in child mortality (under five years of age) at multiple intervals following the launch of a national program. The reduction in child mortality was 36% after 9 to 21 months [24], 77% after 3 to 4 years [32], and 33% after 6 to 9 years [33]. Another RCT in The Gambia showed a 61% reduction in child mortality 0 to 12 months after CHWs began distributing bed nets [29]. Contrarily, a cluster RCT in northern Ghana found a 14% increase in child mortality which was driven by a 135% increase in 1 to 2 year old mortality 5 years after the roll out of the trial [34].

In addition to the limited pool of evidence on the health impacts of CHW programs in sub-Saharan Africa, few programs or process assessments have been conducted. A comparative case study conducted by WHO, using a CHW program functionality assessment tool, found Ethiopia’s Health Extension Program to be functional (score 29/36), while both Uganda’s Village Health Teams (score 20/36) and Mozambique’s *Agentes Polivantes Elementares* (score 19/36) were deemed not functional [19].

In addressing our second aim, if programs are implemented appropriately, the evidence suggests that both generalist and specialist CHWs could be a suitable cadre for engaging adolescents on favorable grounds and potentially facilitating the uptake of adolescent health messages. In most generalist and specialist programs, CHWs spend their time engaging with heads of households. It seems feasible that CHWs, with a reasonable workload and with appropriate training regarding adolescent rights, would also be able to engage adolescents in confidence. This would allow the CHW to: 1) explain to the adolescent the services offered by the YFHS clinic; 2) distribute information about healthy behaviors such as diet, lifestyle, substance misuse, mental health, and sexual/reproductive health; 3) create space for dialogue that enables adolescents to ask the CHW questions or even to discuss an embarrassing condition. In this way, CHWs could potentially use their credibility within the community to build a rapport with a large cohort of adolescents.

Generalist CHW programs could incorporate skills to promote and address adolescent health at the household level. Though the effectiveness of these generalist CHWs is mixed, universal attributes have been identified that would accommodate adolescent health skills [19]. Generalist CHWs are typically selected from their communities by a village health committee, a district health official, or by some combination of the two. Generalist CHWs are over the age of 18, literate, and are often female, though some programs have experimented with all-male CHWs [35,36]. The length of training can vary. For example, Ethiopia’s Health Extension Workers receive six to twelve months of training with frequent refresher training, while Uganda’s Village Health Team CHWs only receive ten days of initial training with refresher training arising as needed [19]. All generalist CHW programs involve a mixture of didactic and skills-based components. Supervision is usually by a health worker from an affiliated primary health center. Supervisory visits tend to consist of site visits, document review, and equipment inventories conducted at least every six weeks. Generalist CHWs are responsible for delivering preventive and therapeutic services [19], such as community mobilization through health education, water, sanitation and hygiene like in The Gambia [33], or dressing wounds, treating uncomplicated cases of childhood diarrhea and malaria, and referring acute conditions to local health facilities as in Burkina Faso [36]. Almost all generalist CHWs are loosely connected to a primary health facility/post, but most of their time is spent moving from house-to-house or organizing community events. In some programs, like Ethiopia’s Health Extension Program, generalist CHWs are salaried, fully recognized members of the health workforce, whereas in many other programs, they are volunteers accountable only to members of the community in which they serve [19].

Alternatively, specialist CHW models suggest that strategies to develop a new cadre of CHW devoted exclusively to YFHS may warrant consideration. Also, the findings indicate that the number of articles reporting specialized CHWs for malaria is higher in comparison to other conditions, which may be reflective of health systems in sub-Saharan Africa. There is, however, considerable variation across the different types of specialist CHWs reviewed. Consequently, it may be useful to focus on one type of specialist CHW, such as that of the nutrition CHW. The recruitment criteria and profile of specialist CHWs in nutrition programs is typically the same as for the generalist CHWs mentioned above. In nutrition programs, training occurs via a similar mix of didactic and practical components, but on average, training time periods are shorter, from three to four days for community volunteers in the Democratic Republic of the Congo [37] and Nigeria [38], to over one month for ‘mother mentors’ in South Africa [39]. The primary difference between specialist nutrition CHWs and generalist CHWs is the scope and quantity of tasks they are expected to execute. Nutrition CHWs can focus on preventive practices such as breastfeeding counselling, child growth surveillance, malnutrition screening, dietary advice, family resource management [40], health education seminars, community gardening demonstrations [41], promotion of health-seeking behavior for pregnant women, and coordinating referrals to other healthcare providers [42]. Although, like generalist CHWs, some specialist programs build upon the initial contact with the health system to provide social services that indirectly affect health. For example, in South Africa, ‘mother mentors’ provided referrals for mental health issues, partner abuse, or legal problems as well as ensuring that mothers were utilizing social entitlement programs, understood nutrition and hygiene, and that their children had received up-to-date immunizations and recent deworming treatment [39]. These additional services might be offered in an attempt to increase the credibility of specialist CHWs, who tend to focus on the less visible preventive and promotive, as opposed to curative, aspects of community health. Despite these occasionally broader nutrition programs, specialist CHWs are commonly supervised, usually once a month, to support the delivery of a core set of nutrition skills. Supervision occurs in much the same way as for generalist CHWs. However unlike generalists, nutrition and other specialized CHWs are rarely paid and are often considered peripheral to the health workforce [19].

While both generalist and specialist CHWs are possible avenues for strengthening the delivery of YFHS, there is not enough evidence to support one approach over the other. To the best of our knowledge, no study has looked at the comparative effectiveness between generalist and specialist CHWs. For either approach to prove viable for delivering adolescent health services in sub-Saharan Africa, a sizable degree of experimentation should occur. Thus, the decision to add adolescent health skills to existing generalist CHWs or to create a new cadre of specialist adolescent CHWs is likely to depend on context-specific features of health systems such as administrative capacity, cost, and political support. How these programs are implemented should form an important line of research and findings could be embedded into decision-making processes [43]. In this way, a body of evidence will emerge to support the adoption of either generalist or specialist CHWs to strengthen YFHS in sub-Saharan Africa.

### Limitations

There are five limitations to this study. First, we attempted to simultaneously conduct a literature review on CHWs in sub-Saharan Africa while examining the concept of ‘generalist’ and ‘specialist’ CHWs for the delivery of adolescent health services. This posed difficulties for a review article with limited space considerations. For example, important questions about the pros and cons of generalist and specialist CHWs remain unanswered. Second, the analytical value of the review was limited by the scarcity of data on the subject. Third, the publication of multiple trials within the same study led to overrepresentation in the literature. For example, several papers were found which corresponded to productive research groups for malaria in The Gambia and neglected tropical diseases in Uganda. Fourth, our search criteria excluded non-English language abstracts, which could have biased the research towards CHW programs in English-speaking countries. Fifth, YFHS, as conceived in the WHO framework, describes qualities that acceptable adolescent services should possess, as opposed to strategies for delivering them. Like others [18], we argue that YFHS is being used to guide service delivery, but the extent to which this is an effective approach in LMICs or consistent with WHO’s intentions is unclear from the literature.

## Conclusions

This paper raises several important considerations for researchers, health workers, and decision-makers about delivering adolescent health services in sub-Saharan Africa. First, if YFHS is to be successful in improving the health and well-being of adolescents in sub-Saharan Africa, innovative approaches to service delivery and outreach, such as those related to CHWs, need to be explored. Second, the quantity and quality of research on CHW programs needs to be increased if this approach is to provide a meaningful contribution to adolescent health services in particular and primary healthcare more broadly in LMICs. In particular, more research is needed on: (i) the characteristics and training of CHWs that may improve the trust relationship, counter stigmatization, and improve effectiveness of the adolescent CHWs, and (ii) rigorous process evaluations that include economic costing data to establish the comparative effectiveness of either generalist or specialist CHW models of adolescent service delivery. Third, decision-makers should partner with academic institutions and engage in existing knowledge translation platforms to ensure that evidence on CHWs and adolescent health is incorporated into the policy process. Finally, if adolescent health services are to be provided at the household level, supportive supervisory structures should be installed to ensure that either generalist or specialist CHWs receive an adequate level of support and are incorporated as formal members of the health workforce.

## Abbreviations

AFHS: Adolescent Friendly Health Services; CHW: Community health worker; HIV: Human immunodeficiency virus; LMIC: Low- and middle-income country; MDG: Millennium Development Goals; NGO: Non-governmental organization; NTD: Neglected tropical disease; PHC: Primary healthcare; RCT: Randomized controlled trial; TB: Tuberculosis; UN: United Nations; WHO: World Health Organization; YFHS: Youth Friendly Health Services.

## Competing interests

The authors declare that they have no competing interests.

## Authors’ contributions

ADK conceptualized the study and wrote the manuscript. SAN and JG conceptualized the study and commented on multiple drafts of the manuscript. ADK, JG, and SAN have read and approve of the final version.

## Supplementary Material

Additional file 1 **The Youth Friendly Health Services framework**[1]. Click here for file
