# Supplementary material for: A review of generalist and specialist community health workers for delivering adolescent health services in sub-Saharan Africa
Source: Hum Resour Health. 2013 Oct 26;11:54. doi: 10.1186/1478-4491-11-54 (PMC3874771; doi:10.1186/1478-4491-11-54)
Supplement: Additional file 1 — The Youth Friendly Health Services framework[1]. [file 1478-4491-11-54-S1.docx]

| **Additional file 1** Features of Youth Friendly Health Services | | |
| --- | --- | --- |
| **1. Adolescent Friendly Policies**  -specify rights  -account for special needs  -non-restrictive care  -gender considerations  -privacy and confidentiality  -free or affordable services | **5. Adolescent Friendly Health Facilities**  -provide a safe environment  -convenient working hours  -offer privacy/avoid stigma  -provide information | **9. Appropriate and Comprehensive Services**  -address physical, social, mental health needs  -comprehensive care package  -only necessary procedures |
| **2. Adolescent Friendly Procedures**  -easy confidential records  -short waiting times  -flexible consultation | **6. Adolescent Involvement**  -well informed about rights  -encouraged to respect others  -involved in service assessment and provision | **10. Effective Health Services for Adolescents**  -evidence-based guidelines  -equipment and supplies  -quality improvement process |
| **3. Adolescent Friendly Providers**  -technically competent  -communication skills  -motivated and supportive  -non-judgmental  -devote adequate time  -act in client’s interest  -treat clients with respect  -provide information | **7. Community Involvement and dialogue**  -promote health services  -encourage parental support | **11. Efficient Services**  -Management information services  -systems of care |
| **4. Adolescent Friendly Support Staff**  -understanding and considerate  -competent and motivated | **8. Community based, outreach**  -outreach workers  -peer-to-peer services |  |

Source [[1](#_ENREF_1)]

1. WHO: **Adolescent friendly health services: an agenda for change**. In*.* Geneva: World Health Organization; 2002.
